# Advancing methodological guidance for audio- and video-based observational studies of communication in nurse–patient encounters: A discussion paper

**DOI:** 10.1016/j.ijnsa.2026.100627

**Published:** 2026-07-10

**Authors:** Annelie J Sundler, Malin Östman, Lena Hedén, Inger K Holmström, Sandra van Dulmen

**Affiliations:** a The Swedish Red Cross University, Stockholm, Sweden; b Department of Health Sciences, Innovation and Design, Mälardalen University, Västerås, Sweden; c Research, Education, Development & Innovation, Primary Health Care, Region Västra Götaland, Sweden; d General Practice/Family Medicine, School of Public Health and Community Medicine, Institute of Medicine, Sahlgrenska Academy, University of Gothenburg, Gothenburg, Sweden; e Faculty of Caring Science, Work Life and Social Welfare, University of Borås, Borås, Sweden; f Department of Public Health and Caring Sciences, Uppsala University, Uppsala, Sweden; g Nivel (Netherlands Institute for Health Services Research), Utrecht, the Netherlands; h Department of Primary and Community Care, Radboud Institute for Health Sciences, Radboud University Medical Center, Nijmegen, the Netherlands

**Keywords:** Audio recording, Communication, Data collection, Nurses, Observation, Observational study, Video recording

## Abstract

**Background:**

Communication is a cornerstone in nursing. Research on nurse–patient communication faces well-known challenges, as encounters are dynamic, context-dependent, and shaped by subtle verbal and non-verbal cues. Capturing this complexity requires rigorous observational approaches, of which audio and video recordings are key methods, enabling documentation of communication as it unfolds in practice. However, collecting such data in clinical settings involves significant methodological and ethical considerations.

**Objective:**

The aim of this paper is to provide guidance for methodological rigor in data collection using audio or video recordings when observing clinical communication in patient encounters.

**Information sources:**

This discussion paper draws on theory and previous empirical research on communication in clinical patient encounters using data collected with audio and video recordings.

**Results:**

The practical guidance is structured into five key areas spanning the data collection process: (1) planning and preparation, (2) audio versus video recordings, (3) ethical and legal concerns, (4) information and recruitment, and (5) procedures for collecting recordings. Each area includes methodological considerations related to the design and conduct of data collection, as well as recommendations for transparent reporting to support rigor.

**Conclusions:**

This guidance supports researchers in addressing key methodological challenges in data collection using audio and video recordings in studies of clinical communication. It highlights considerations relevant to the design and conduct of the research process, including issues of ecological validity and generalizability, and emphasizes the importance of transparent reporting of methodological decisions. In light of previous inconsistencies in reporting, such guidance is essential to strengthen methodological rigor.

By adopting more robust methodological strategies, ethical and scientific standards in study design and data collection can be strengthened.

**What is already known**

• Observational research can provide key insights into communication and behaviour essential to nursing education and daily care, which cannot be captured equally reliable with e.g. interviews.
• Reporting guidelines like STROBE focus on study reporting and provide limited methodological guidance for the ethical and rigorous design of data collection using audio and/or video recordings.

Alt-text: Unlabelled box dummy alt text

**What this paper adds**

• Provides methodological guidance for the ethical and rigorous design of nursing research using audio and video recordings.
• Outlines strategies for managing data collection to support ecologically valid and generalizable observational data.

Alt-text: Unlabelled box dummy alt text

## 1 Background

Communication is a cornerstone in nursing. Therefore, studies of nurse-patient communication are essential to develop best practices (Höglander et al., 2023). However, research on nurse–patient communication in clinical practices faces well-known challenges. While observational research offers unique opportunities to study communication as it naturally occurs in nurses’ clinical practice, collecting audio and video data presents methodological and ethical challenges, as real-life clinical interactions are dynamic, context-dependent, and shaped by subtle verbal and non-verbal cues. Capturing this complexity requires rigorous observational methods that can document communication as it unfolds in practice. Scientific rigor is defined as the systematic and transparent application of research methods, including the data collection, to ensure robust, unbiased, and well-controlled design, and reporting (National Institutes of Health, 2024). Rigor refers to the practices that make the research process systematic and transparent, strengthening the robustness of the findings. Despite the growing use of audio and video recordings in nursing and healthcare research, there remains limited guidance on how to plan and conduct such data collection in ways that ensure methodological rigor. This paper aims to address this gap by advancing the methodological guidance in this area by outlining key considerations and practical strategies for researchers undertaking data collection with audio and video recordings of nurse–patient communication. While the focus is on nurse–patient interactions, the guidance is also relevant to observational studies of communication involving other healthcare professionals.

### 1.1 Communication in the context of nursing research and practice

Communication and relationships are fundamental in nursing care because the interaction between the nurse and the person receiving care is central to delivering person-centred care (McCormack and McCance, 2006; Kitson et al., 2010; Dewar and Nolan, 2013). Empathy, respect for patients’ autonomy, and understanding their needs and perspectives all require good communication. Communication skills and a person-centred approach can enable collaboration, shared decision-making, and care that is tailored to the individual.

Communication research in nursing is particularly important for supporting the development of person-centred communication skills that enhance patient well-being and improve care quality. In addition, ontological assumptions underlying nursing research methods reflect the relational and interactional nature of nursing care. For example, grounded theory, which is often associated with social constructionism, emphasises the co-creation of meaning through social interactions (Corbin and Strauss, 2008). Phenomenology, drawing on the lifeworld perspective and human intersubjectivity, focuses on shared experiences and mutual understanding within relational contexts (van Manen, 1997). These approaches underscore the interpersonal dynamics in advancing nursing research and practice. Moreover, nursing practice is complex: care is provided in place, during transitions, across various environments, and while performing a range of tasks. In these contexts, communication serves multiple functions simultaneously, for instance as social, informative, and supportive, as illustrated by the COMCARE model (Sundler et al., 2025).

Although health communication research has made considerable progress in recent decades (Finset et al., 2023), the scope of empirical communication research in nursing remains less broad than that of physician observational studies (Höglander et al., 2023). Audio and video recordings of real time patient encounters is widely used across different research fields. A recent review of video-based observations in healthcare found that most studies (81% of 175) focused on patient–clinician communication, and only 39 of these included nurses (Golembiewski et al., 2023). To advance this field, there is hence a need for further research aimed at understanding effective communication strategies and training in nurse–patient interactions. In line with Golembiewski et al. (2023), we argue that such research requires methodological guidance to ensure rigor and impact. Moreover, our own research experience has highlighted the lack of such methodological support, further underscoring the need for clearer guidance in studies using audio and video recordings.

The contribution of observational methods to communication research in nursing practices lies in their ability to provide rich, contextual insights into complex phenomena and dynamics that may be difficult to articulate and can differ from views expressed in interviews, which can be influenced by social desirability bias. Additionally, they are suggested as helpful in uncovering taken-for-granted aspects of situations and in including individuals who might otherwise be excluded due to perceived research burden (Walshe et al., 2012). While the use of observational methods in research is not new, they are particularly well suited for studying naturally occurring behaviours and interactions in healthcare practices (Fix et al., 2022). Catchpole et al. (2017) similarly emphasise that observations allow researchers to capture *what really happens* rather than rely on perceptions or assumption.

### 1.2 Examples of audio- and video-based studies informing nurses communication

Empirical communication research using audio and video recordings in nursing practice has been conducted in various clinical settings and with different patient groups (Höglander et al., 2023). Examples include emotional communication in home care with older persons (Höglander et al., 2020) and in cancer care (Uitterhoeve et al., 2009), children’s emotional distress during preoperative needle procedures (Kleye et al., 2022), communication on concerns during telephone nursing calls (Ernesäter et al., 2016), and nursing support in home hospice care (Clayton et al., 2017). Taken together, this research highlights challenges in aligning communication with patients' individual needs and preferences.

Research using audio and video recordings provides valuable insights into nursing communication, for instance how communication in nurse–patient encounters require a careful balance of medical, nursing, task-oriented, and affective aspects to ensure person-centred care and promote health (Höglander et al., 2023). Such studies have revealed how emotional, relational, and interactional dynamics shape care delivery in ways that are not always visible in self-reported data or field notes. In another study Gustafsson et al. (2025) investigated emotional communication in home care encounters with older persons, showing that recorded interactions reveal subtle emotional cues and strategies essential for supporting person-centred care in challenging situations. These insights are critical for improving communication practices because recordings help reveal mechanisms of supportive communication, identify barriers to patient engagement, and contribute to evidence-based improvements in clinical practice, particularly in settings characterized by time constraints and diverse patient needs.

### 1.3 The value of observations using audio and video recordings

Observational research using audio or video recordings includes the collection and analysis of communication behaviours. This paper focuses specifically on the methodological considerations related to the *collection* of audio and video recordings. These data allow researchers to investigate communication patterns and mechanisms as they unfold in patient encounters, capturing aspects that participants may not be fully aware of or may not recall in interviews (van Dulmen et al., 2012; Golembiewski et al. 2023). Importantly, recordings document routine clinical interactions as they occur, providing authentic evidence while avoiding the additional demands on patients and staff associated with follow-up interviews or more intrusive observational protocols. Audio and video recordings allow for repeated reviewing and detailed examination of multiple behavioural and contextual aspects (Williams et al., 2013; Asan and Montague, 2014), making it possible to explore links between specific conditions or events, as well as identify preceding factors and their subsequent outcomes.

Methodological guidance is essential for gaining valid and reliable insights into communication practices during everyday healthcare encounters, as well as related ethical aspects and considerations, to provide knowledge that informs nursing education, practice, and healthcare policy. A range of existing guidelines offers support for different aspects of observational methods—for instance, participatory or direct observations (Catchpole et al., 2017; Fix et al., 2022), systematic reporting of studies that code communication and behaviours (van Dulmen et al., 2012; Hillen et al., 2025), the use of video in primary care research (Asan and Montague, 2014) and strategies to improve the reliability of video recordings (Haidet et al., 2009). Communication ratings from audio versus video data have also been compared (Williams et al., 2013). Building on this existing work, the present study advances the field by addressing methodological gaps in the design and conduct of data collection using audio or video recordings, with a particular focus on supporting rigor and transparency in clinical communication research.

### 1.4 Challenges and methodological gaps related to observations using audio and video recordings

Conducting and reporting observational studies using audio and video recordings presents numerous methodological and practical challenges. A recent review by Golembiewski et al. (2023) identifies several gaps that motivate the need for further methodological guidance. These include inconsistent reporting of design and ethical features, limited justification for choosing video versus audio despite their different methodological implications, and the continued underuse of video in healthcare research even though it has strong potential to illuminate care processes. Key considerations include the researcher’s role, informed consent, ethical and privacy concerns, as well as potential issues related to subjectivity and bias (Catchpole et al., 2017; Walshe et al., 2012). Because audio- and video-based observations allow researchers to engage with participants and settings in more or less active ways, these decisions must be made and reported transparently (Asan and Montague, 2014; Haidet et al., 2009). Together, these challenges underscore the need for methodological clarity to support rigorous decision-making and enhance the quality of reporting in studies that use audio or video recordings. Hence, the aim of this paper is to provide guidance for methodological rigor in data collection using audio or video recordings when observing clinical communication in patient encounters.

## 2 Methodological guidance for data collection using audio- and video-based observational research

This section presents practical methodological guidance structured into five key areas spanning the data collection process. This paper is drawing on theory and research, including the authors’ extensive experience in communication studies using observational audio and/or video recordings across all age groups—from children to older adults. Furthermore, experts in the field have been consulted when developing these strategies into the identified key areas. In the following sections, each key area is elaborated, including methodological considerations related to the design and conduct of data collection. Table 1 presents the key areas, a brief description of their scope, and the corresponding methodological considerations that should be addressed and transparently reported to support rigor.

**Table 1:** Overview of key areas, their scope, and associated methodological considerations for data collection and reporting in audio- and video-based observational communication research.

Key Areas	Focus of each key area	Methodological considerations to be addressed and reported
1. Planning and preparation	**Study design and rationale** – defining objectives and research questions, justifying the use of observational methods, and deciding between audio or video recordings (e.g. capturing verbal, paralinguistic and non-verbal). **Setting, participants, and situations** – specifying the study context, participants, and communication situations, and addressing practical considerations such as timing, equipment, and placement.	What are the study objectives and research questions? Why are observational data using audio or video recording necessary to address these questions? What aspects of communication (verbal, paralinguistic, non-verbal, contextual) are being explored? What is the study setting, sample and communication situations included?
2. Audio versus video data	**Comparing quality, advantages, and limitations of audio and video recordings** – including the types of communication captured (verbal versus non-verbal), as well as practical and contextual aspects such as intrusiveness, manageability, and environmental factors.	How were audio and video methods considered in relation to the research questions? What are the strengths and limitations of each method in the context of the study? How do the chosen recording methods influence the type and quality of data captured?
3. Ethical and legal concerns	**Informed consent, confidentiality and privacy** –including procedures for obtaining consent, providing clear information to participants, safeguarding confidentiality, and ensuring data-security. **Non-maleficence and integrity** – including avoiding harm, ensuring transparency and upholding ethical standards in data collection.	How were informed consent procedures conducted? Was ethical approval obtained? How were privacy, confidentiality, and participant identification or de-identification managed? Were any adaptations made for sensitive contexts or participant groups? How were data stored, and how were ethical and legal requirements addressed in the study design and data collection procedures?
4. Information and recruitment	**Information provision and transparency** – ensuring clear communication about the study aims, procedures, and expectations for participants. **Recruitment processes and participant engagement** – including strategies to enhance motivation, acceptability, trust, and respectful interaction with participants.	How were participants informed about the study aims, procedures, and use of audio or video recordings? How were participants recruited, and what strategies were used to support engagement and acceptability? What were the participation rates, and how were they calculated (if relevant)? What reasons for non-participation were identified, if any?
5. Procedures for collecting recordings	**Sampling and inclusion of participants -** including eligibility, inclusion and exclusion criteria, and considerations related to sample size. **Recording procedures and researcher role** – including the choice and placement of equipment, as well as the level of researcher involvement and observer role during data collection.	What were the inclusion and exclusion criteria, and how were participants selected? What was the rationale for the sample size, including the number and duration of observed sessions or situations? What equipment was used, and how was it positioned during data collection? What was the researcher’s role during data collection, and what level of involvement or observer presence was applied?

### 2.1 Planning and preparation

Planning and preparation involve defining the scope and design of the study and ensuring that practical conditions enable successful data collection.

#### 2.1.1 Study design and rationale

Outline your study focus by clearly defining the objectives, research questions and context to ensure that the observational data collected is accurate and meaningful. The choice between audio or video recording should be guided by the type of communication and interaction of interest, as well as the level of detail and richness required for analysis. At the same time, the characteristics of the patient group (e.g., degree of illness, communication abilities, sensitivity of topics discussed) and the clinical setting (e.g., level of privacy, care intensity, institutional regulations) may limit or favour recording methods, shaping what is ethically and practically achievable. Yet studies often describe these decisions only brief and without an explicit rationale (Golembiewski et al. 2023). Clarifying these ensures alignment between the study design, data collection strategy, and analytic approach, thereby strengthening rigor and relevance.

The rationale for using observational data should be explicitly linked to the research questions and the specific forms of communication or interaction that the study seeks to examine. Depending on the study’s focus, different communicative dimensions may be relevant:

• **Verbal communication** – the words and content of speech
• **Paralinguistic communication** – vocal qualities such as tone, pitch, pace, and pauses
• **Non-verbal communication** – bodily and physical behaviours such as gestures, posture, gaze, and spatial orientation

Articulating which of these dimensions are essential and why, provides justification for selecting either audio or video recordings and supports methodological transparency throughout the study.

The objectives and research questions guide both the data collection and the choice of method for analysis. Depending on if the aim is to measure specific variables, explore patterns, gain in-depth insights, or generalize findings across contexts or populations will determine whether data collection needs to be aligned with quantitative, qualitative, or mixed methods designs and methodologies. The data collection process plays a pivotal role because it influences the validity, reliability, and interpretability of the findings.

To show how research design shapes data collection, we present two contrasting studies with different objectives. The examples are from the COMHOME project (Hafskjold et al., 2015), investigating communication between older persons and nurses in home care settings. The first study used a cross-sectional design exploring patient-centred aspects of home care communication. Research questions on quantitative nature were related to patient-centeredness and whether patient-centeredness was impacted by characteristics of either the older persons, nurses or visits. While patient-centeredness was coded with the Roter Interaction Analysis System (RIAS), a generalised linear mixed model was used for the statistical analysis exploring the influence of characteristics on the degree of patient-centeredness (Höglander et al., 2020). In another study, a qualitative approach was used to explore person-centred communication in a selection of 16 audio recorded home care visits. To gain in-depth understanding of person-centred communication attributes in conversations during home care visits with older persons, the selected audio recorded data was analysed using a method for qualitative thematic analysis (Sundler et al., 2020).

#### 2.1.2 Setting, participants and situations

The next step in the planning process is to decide the study setting, the participants, and the types of situations to be observed. To ensure successful data collection, researchers need to set realistic and context-appropriate goals. This can be supported by systematically considering *what* will be recorded, *where* the observations will take place, *when* data collection will occur, and *how* the recordings will be carried out, see Table 2.

**Table 2:** Detailed guidance for the planning phase of observations (what, where, when, and how), as part of the overall data collection process in audio- and video-based observational communication research.

	Description	Examples
*What?*	**Situations** to be observed.	Specific consultations, medical or nursing procedures (e.g., wound care, needle procedures), patient education sessions, home-care visits.
*Where?*	**Setting** in which the observations will take place. Availability of situations with relevant patient/provider interactions. Plans for seamless and unobtrusive observations. Negotiating with stakeholders.	Hospital care, primary care, prehospital care, elderly care.
*When*?	**Timing** of data collection to ensure access to relevant situations and to minimize disruptions.	Ultimate length of situations, specific procedural times, avoiding shift-change periods.
*How*?	**How** recordings will be carried out, including roles, technical setup and preparation, steps to minimize intrusiveness.	**Operational model** - managed by researcher, healthcare professionals, both or others? **Monitoring** - actively monitored (start/stop, quality checks) or unmanned/automated. **Equipment suitability** - Wireless vs. wired devices, battery life, storage capacity. **Placement** - fixed vs. mobile devices, angles to capture interaction while minimizing intrusiveness, mic or camera placement for optimal recording. **Technical preparations** - functionality checks, gain levels, time/date sync, file naming conventions. **Piloting** - testing procedures and equipment in the actual environment to refine workflow and positioning. **Backup plans** - batteries, secondary recorder. **Data handling at site** - immediate encryption needed, secure transfer, access controls. **Indicators & ethics** - visible recording indicators, pause/stop protocol on request.

Securing field access is a key preparatory step for conducting audio and video recorded observations and typically involves negotiating with relevant stakeholders. Drawing on our experience of both successful and challenging data collection processes, researchers should identify key stakeholders and other relevant personnel to obtain their cooperation and ensure access to the settings where data collection will take place. Establishing this collaboration early allows researchers to clarify expectations, define roles, and address potential concerns related to workflow, privacy, or ethical requirements.

Make contingency plans on how to manage potential technical failures and environmental disruptions. Good preparation prevents avoidable disruptions and data-quality issues; poor planning risks logistical difficulties and compromised validity.

Studying a range of clinical settings, including complex care and unplanned encounters, is important to ensure that findings reflect nurses’ real practice and can inform care delivery and education. Challenges in these environments should be viewed not as barriers but as factors requiring thoughtful planning (Broyles et al., 2008). Because no single approach fits all situations, each study must consider its specific setting, participants, and available time and resources. Flexibility and adaptability are essential for selecting methods that best suit the circumstances.

### 2.2 Audio versus video data

#### 2.2.1 Comparing quality, advantages, and limitations of audio and video recording

Audio and video recordings each have their advantages and limitations in communication research. Audio recordings capture verbal and paralinguistic aspects of communication, such as words, tone, pacing, and responsiveness, but are limited in conveying non-verbal cues. Video recordings offer visual cues, such as facial expressions, gestures, and body movements providing a more comprehensive picture of nurse-patient interaction. Yet, audio recordings have been shown to effectively capture the emotional aspects, with strong correlations found between audio and video data (Williams et al., 2013). While video-based data offer non-verbal details, audio-based data can still be efficient in understanding of emotional and relational dynamics in nursing care.

Audio recordings are generally perceived as less intrusive, more cost-effective, and easier to manage, making them especially appropriate for studies involving vulnerable populations (Williams et al., 2013). They are also easier to set up and are often more acceptable to participants. In addition, audio recordings can be started and stopped more unobtrusively; however, this also requires careful attention to ensure that recording does not begin too late or end too early, as this may result in missing important contextual cues. Although audio recordings do not capture visual non-verbal communication, they may help reduce certain ethical and practical challenges without significantly compromising data quality, supporting their broader use in healthcare communication research.

Video recordings can present logistical and ethical challenges (Asan and Montague, 2014). In our experience, they may raise additional privacy concerns compared to audio recordings and require more extensive setup and careful planning related to framing, distance, angle, and other visual considerations. Video recordings may also be perceived as more intrusive, as the presence of a camera can create discomfort for both patients and nurses. Similar to audio recordings, important non-verbal sequences may be missed if the camera is turned on too late, or the camera is placed in a wrong angel or corner. Depending on the situation, it can also be challenging to ensure that unintended individuals are not captured in the background. The sense of intrusiveness often stems from participants’ own concerns. For instance, in one of our studies, children expressed privacy worries and feared that video recordings might be shared on social media. Moreover, participants may feel vulnerable in certain situations—such as during physical examinations or other private moments—where video recordings can heighten feelings of exposure and intrusion.

Ensuring high-quality data includes selecting equipment with the necessary audio clarity and/or visual resolution to minimise data loss or misinterpretation related to equipment problems. Advancements in technology have led to the development of smaller, lighter devices, enabling discreet usage and enhancing usability in clinical practices. Moreover, proper camera or audio recording positioning is vital to capture relevant interactions and communication without being overly intrusive or distracting, while also minimising disturbances from background noise in the environment. The training of researchers or healthcare professionals to handle equipment and troubleshoot issues are also essential for maintaining data quality.

Creating a comfortable and as natural an environment as possible for both patients and nurses, whether using audio or video recording, can help support natural, real-time communication and encourage participation, thereby enhancing the overall quality of the data collected. A main concern in the literature is how the recording process may influence communication. However, Parry et al. (2016) argue that it is not possible to entirely exclude the potential effects of being recorded, even though these effects do not appear to adversely impact healthcare interactions or communication behaviours. Although participants remain aware of being recorded, and generally do not forget it, most patients who were audio or video recorded reported feeling only slightly, or not at all, influenced by the recording. Still, it remains important to take precautions to make the recording process as unobtrusive as possible. For an overview of pros and cons with audio and video recordings, see Table 3.

**Table 3:** Overview of differences between audio and video recordings.

	Audio recordings	Video recordings
Study design implications	Captures verbal/spoken words and some paralinguistic elements; useful for studies focused on content or conversation patterns	Captures verbal and paralinguistic communication, and non-verbal behaviour; chosen for interactional and embodied communication
Preparation workload	Similar, but may be easier to plan (one recorder placement)	Similar, but may need some more planning related to the environment, camera angel, lighting, privacy
Burden on participants	Less intrusive	Visible camera – may lead to more reactivity
Feasibility in clinical settings	Device placement for sound, near speaker and sensitive to background noise	Must capture the scene and is sensitive to light, movement, visual obstructions
Failure risks	Sensitive to noise, mic blocked	Sensitive to space, lighting and visibility, angel wrong, people blocked, and privacy
Privacy risks and vulnerable situations	Moderate and lower impact	Higher privacy risks and ensure no unintended people or sensitive objects appear in the frame, may feel more intrusive
Acceptability and sample size	Higher acceptability and easier to recruit participants	May lower acceptability as people may worry about being filmed, may be more participants that decline

### 2.3 Ethical and legal concerns

Ethically and legally sound research processes are essential when conducting real time recordings of communication in healthcare settings. Data collection processes must fulfil with the relevant ethical guidelines and legal requirements in the country in which the research is conducted.

#### 2.3.1 Informed consent, confidentiality, and privacy

Ethical considerations in audio- and video-based observational research include the responsibility to obtain valid informed consent, provide clear information to participants, protect identifiable data, particularly in video recordings, and ensure robust safeguards for confidentiality, privacy, and secure data storage and sharing. Before the start of the data collection, ethical approval must be obtained from the relevant institutional review boards or ethics committees. Ethical use requires considerations surrounding patient privacy, ownership of recordings, patient autonomy and healthcare provider responsibilities, as well as close collaboration among researchers, nurses, and other stakeholders to address these concerns effectively (Zainal Abidin and Razali, 2024).

Most people consider video-based research acceptable and valuable, although it still carries certain risks (Parry et al. 2016). Patients need to feel comfortable enough to not withhold sensitive information due to privacy concerns. This includes security measures that require audio and video data to be encrypted to protect confidentiality and prevent the identification of participants (Williams et al., 2013).

To address confidentiality, ensure that both patients and nurses understand the research process, feel reassured about their privacy, and are treated with respect. Explain how data will be securely stored, who will have access to them, and the measures in place to maintain participants' anonymity. Informed consent must be obtained voluntarily, and participants should know that they can withdraw at any time without consequence. To safeguard confidentiality, all data used must be anonymised to protect participants' identities and sensitive information, and access to recordings should be restricted to authorised researchers only. Parry et al. (2016) point to the inherent lack of anonymity in video recordings and the absence of consensus on how to de-identify such data. Even when images are de-identified, their use and display can be difficult to control and may lead to unforeseen risks. Moreover, little is known about how these challenges may evolve in the future as recording technologies and data-sharing practices continue to develop. Importantly, similar concerns may also arise in audio recorded data, where specific locations, care routines, or situational details mentioned in conversation can make the context, and potentially even the individuals involved recognizable to others, despite the absence of visual information.

#### 2.3.2 Non-maleficence and integrity

Ethical considerations here concern researchers’ responsibility to minimize harm, avoid unnecessary intrusion or disruption, and conduct observations with transparency and integrity. These principles guide decisions about how observations are planned, implemented, and reported. Researchers must be well-versed in research ethics and remain committed to upholding ethical standards. This includes ensuring that ethical principles are not compromised and that research processes align with the values of honesty, respect, and accountability, and minimising psychological discomfort.

Transparency in data collection is essential to ensure scientific integrity and to prevent fabrication, falsification, or misrepresentation of data. In line with the FAIR principles (Wilkinson et al., 2016), researchers should establish procedures that make data findable, accessible, interoperable, and reusable, including clear protocols for data sharing, storage, and future use. At the same time, ethical requirements stipulate that access to identifiable data must be restricted to authorised members of the research team only. Data storage includes clearly documenting how data are collected, managed, and verified, as well as providing accurate reporting and appropriate acknowledgment of sources. Such practices support trust, reproducibility, and responsible data stewardship throughout the research process. National regulations governing research ethics vary, requiring researchers to navigate differing legal and ethical requirements across countries

### 2.4 Information and recruitment

#### 2.4.1 Information provision and transparency

Approach participants well. Provide clear and transparent information prior recordings. Explain the aims, procedures and use of recordings to support informed and confident participation. Allow time for participants to ask questions and make an informed decision. In line with Parry et al. (2016), we strongly recommend providing information and approaching patients for consent in plain language, understandable for all before the healthcare encounter is recorded. Consent should be obtained as far in advance as possible to allow time for questions and to avoid situations in which recordings must be made with little notice. Provide information verbally and in writing, and provide materials with clear information and plain language, if relevant, ensuring accessibility for diverse populations.

Make sure participants understand what their participation involves and how the recordings will be used, as well as their right to withdraw. For acceptability, it may be important to emphasise that the aim is not to evaluate individual performance but to explore patterns of communication across multiple recordings and participants. Although individual actions may be observed, the primary focus lies in identifying common trends and behaviours that emerge across different individuals or contexts.

Consider the most effective methods and timing for providing information to the target group. Ensure that communication about the research is clear and highlights potential benefits for improving healthcare practices, as this can help foster positive perceptions. While nurses are commonly informed directly by researchers through workplace meetings and written materials, patient information can be delivered through various channels, including direct invitations from nurses or researchers, postal mail, or social media.

#### 2.4.2 Recruitment processes and participant engagement

Researchers can influence participants’ willingness to take part in research. Building trust and fostering positive attitudes toward the study can enhance the motivation and acceptability of being recorded. Showing appreciation for participants’ time and involvement reinforces the value of their contribution. Treating participants respectfully helps create a sense of safety and comfort during the research process. The presence of a camera can elicit questions about who will have access to it and how it will be used.

Overall, professionals, patients, and their families are generally positive toward participating in recordings. Some studies report that patients are less concerned than clinicians about being video recorded (Scott et al., 2020). In sensitive contexts the added value of video must be carefully weighed, as concerns about confidentiality are often more pronounced with video data. Researchers should avoid approaching participants in ways that make them feel scrutinised or pressured.

Offering incentives, such as financial compensation, access to additional resources, or tokens of appreciation, can increase willingness to participate, particularly among patients. However, cultural differences may influence what is considered appropriate in different countries, and Ethical review boards might have different approaches to compensation.

### 2.5 Procedures for collecting the recordings

#### 2.5.1 Sampling and inclusion of participants

In addition to determining an appropriate sample size, it is essential to identify eligible participants and define inclusion and exclusion criteria aligned with the research question. These criteria should be sufficiently specific to ensure meaningful results, yet not so restrictive that they limit the relevance or feasibility of the study.

Although sampling is a critical methodological concern, discussions of sample size are notably scarce in the existing literature on audio- and video-based observational research, where reporting of key methodological elements is often inconsistent (e.g., Golembiewski et al., 2023).

In qualitative approaches, such as thematic analysis or conversation analysis, sample size is typically guided by the principle of data saturation, with the aim of achieving depth rather than breadth. This often involves a smaller number of recordings for in-depth analysis. In contrast, quantitative approaches require larger samples to ensure statistical power and generalizability, with size determined by factors such as effect size, variability, and unit of analysis (e.g., interactions, participants, or coded behaviours). Practical considerations, including recording length, transcription workload, cost effectiveness and ethical constraints, also influence sample size. Transparency in reporting the rationale for sample size is essential.

In qualitative studies, a sample size of around 15–30 participants is usually sufficient to generate in-depth insights through recorded observations (Hafskjold et al., 2016; Kristensen et al., 2017). Quantitative studies, on the other hand, typically require larger samples to support statistical analysis and ensure generalisability (Sundling et al., 2020).

One strength of using recordings is their potential to include participants who might otherwise be unable or unwilling to take part. Using recordings can broaden participation by reducing the cognitive, emotional, and time demands typically associated with interviews or surveys. Individuals who may find it difficult to articulate their experiences verbally, such as young children, patients experiencing stress or fatigue, or those with communication or cognitive challenges, can still be included, as recordings capture naturally occurring interaction without requiring active verbal engagement in a research dialogue. For example, in studies exploring emotional expressions in child-nurse communication during needle procedures, children as young as six years old have been included (Kleye et al., 2022). Recordings may, when minimally intrusive and well-integrated into clinical workflows, fit more seamlessly into routine practice, thereby enabling participation from nurses and patients who might otherwise decline due to time constraints or discomfort with interview settings.

#### 2.5.2 Recording procedures and researcher role

The choice between audio or video recordings can influence the level of researcher involvement. Video often requires the researcher to be present to operate equipment, while audio is easier for healthcare professionals to manage. In settings like home care, having staff handle recordings may reduce disruption, though it can also add to their workload. The equipment used should, as far as possible, avoid disrupting the flow of care or compromising patient comfort. Whenever feasible, schedule observations at times that are convenient for both patients and healthcare professionals to minimise additional stress. Ensure seamless integration of the recording process into the clinical environment and workflow, and consider how participants are to be involved.

If healthcare professionals are responsible for managing recordings, provide them with adequate training on how to manage the equipment and how to handle situations where a patient may feel uncomfortable or hesitant about the recordings. Also, ensure technical support if needed to make the recording process easy to manage. Strive to normalise the recording process by selecting portable, user-friendly equipment and scheduling recordings at convenient times for all parties involved. The observer role of the researcher, and the clear recording processes (standardized start/stop instructions) help ensure that recording conditions are consistent, and could minimize interruptions, biases, and observer effects.

## 3 Ecological validity and generalisability in audio- and video-based communication research

This discussion advances methodological guidance for conducting observations using audio and video recordings in communication research. It offers practical and reporting recommendations grounded in relevant theory and empirical work. Given ongoing inconsistencies in reporting (Golembiewski et al., 2023), there is a clear need for explicit standards of rigor. Accordingly, researchers should justify key decisions made during data collection and report them transparently, as summarised in Table 1.

One of the primary advantages of audio and video recorded observations is their strong ecological validity. Because data are collected in real clinical settings, they offer insight into communication as it occurs within its natural context, rather than as perceived or experienced by the participants. A potential limitation, however, is reactivity: participants may adjust their communication or become less spontaneous when aware of being recorded (Pringle and Stewart-Evans, 1990; Zhou et al., 2010). Some may also withhold sensitive information due to privacy concerns or hesitate to discuss certain topics openly. In our experience within nursing and healthcare, participants typically acclimate to being recorded within minutes and resume natural interaction. Moreover, the press of clinical work, especially for nurses managing multiple tasks under time constraints, often reduces attention to the recording, further diminishing any sustained reactivity. Taken together, these considerations suggest that while initial reactivity is possible, its effects are usually transient and limited, and the ecological validity of audio- and video-based observations remains a key strength.

A risk with using recordings for data collections is technical constrains such as data loss due to equipment malfunction, or if participants move out of the camera's frame. Managing and storing large volumes of video files also requires robust security and technical infrastructure. While video recordings capture a lot of detail, it only records a partial view of a situation, and the broader context or events occurring outside the camera's focus might be lost.

There are multiple ways to analyse interactions and communication in patient encounters: quantitative (e.g. counts of behaviours or coding of frequencies/durations), qualitative (in-depth thematic or content-based exploration of patterns), or mixed methods (Parry et al., 2016). One practical limitation is the time-consuming nature of data preparation. Some approaches require detailed verbatim transcription (e.g., conversation analysis), whereas others rely on direct visual analysis of video (e.g., sequential interaction coding) and may not require full transcripts. Although recent advances in AI-supported transcription tools (such as Whisper and Amberscript) are beginning to streamline the transcription process, they do not remove the need for researcher oversight, especially in analyses where precision, contextual interpretation, and fine-grained interactional detail are essential. Regardless of approach, analysis remains labour-intensive, involving the coding and interpretation of both verbal and non-verbal cues and the risk of “data overload”. A comprehensive discussion of transcription practices and analytic tools (e.g., conversation analysis, qualitative thematic analysis, RIAS, VR-CoDES, OPTION) is beyond the scope of this paper; however, these approaches are essential for capturing nuance and ensuring validity in healthcare communication research.

## 4 Conclusion

This guidance can support researchers in managing complex data collection and addresses issues of rigor, ecological validity, and generalizability related to audio and video recordings, ensuring ethical and methodological standards throughout the design and data collection process. Transparent reporting is essential for assessing how findings can be applied and generalized to other settings or situations.

To succeed with observational research using audio and video data, researchers should anticipate potential challenges and prepare contingency plans. This includes establishing clear consent and privacy protocols, minimizing observer effects through unobtrusive recording, ensuring backup equipment and secure data storage, and defining procedures for unexpected disruptions.

With robust strategies, audio and video recordings can provide valid and reliable insights into nurse–patient communication practices, enabling evaluation of treatment policies, inform the design of clinical interventions, and provide feedback to nurses. When combined with other sources, such as patient outcome measures, questionnaires, or reflective practice interviews that explore facilitators and barriers to high-quality communication, recordings offer unique insights into everyday clinical practice. Importantly, because they document routine interactions as they unfold, recordings generate rich data with minimal additional burden on patients or professionals, avoiding the extra demands associated with follow-up interviews or more intrusive observational methods, with clear relevance for a range of healthcare professionals beyond nursing.

## Declaration of generative AI and AI-assisted technologies in the writing process

During the preparation of this work, the authors used ChatGPT (OpenAI) and Copilot (Microsoft) to improve the readability and language of the manuscript. After using these tools, the authors reviewed and edited the content as needed and take full responsibility for the content of the published article.

## Funding

This research did not receive any specific grant from funding agencies in the public, commercial, or not-for-profit sectors.

## CRediT authorship contribution statement

**Annelie J Sundler:** Writing – review & editing, Writing – original draft, Visualization, Methodology, Investigation, Conceptualization. **Malin Östman:** Writing – review & editing, Methodology, Conceptualization. **Lena Hedén:** Writing – review & editing. **Inger K Holmström:** Writing – review & editing, Conceptualization. **Sandra van Dulmen:** Writing – review & editing, Methodology, Investigation, Conceptualization.

## Declaration of competing interest

The authors declare that they have no known competing financial interests or personal relationships that could have appeared to influence the work reported in this paper.
